# Unexpected Ureter Rupture Causing Abdominal Pain: A Clinical Case Report and Literature Review

**DOI:** 10.7759/cureus.104811

**Published:** 2026-03-07

**Authors:** Ilyass Laaribi, Samia Berrichi, Ikram Zaid, Houssam Bkiyar

**Affiliations:** 1 Intensive Care and Resuscitation Department, Mohammed VI University Hospital, Faculty of Medicine and Pharmacy of Oujda, Mohammed First University, Oujda, MAR

**Keywords:** abdominal pain, case report, spontaneous rupture, ureter, urinary extravasation

## Abstract

Spontaneous ureteral rupture is a rare condition occurring in the absence of trauma or surgical procedures. The most common cause is ureteral obstruction by a urinary stone. Spontaneous rupture without an identifiable cause is even rarer, as the clinical presentation is nonspecific, posing a diagnostic challenge. We report here the case of a patient presenting with acute abdominal pain mimicking a gastrointestinal origin. This article also reviews cases of spontaneous rupture without obvious causes described in the literature over the last 20 years.

## Introduction

Spontaneous ureteral rupture represents an infrequent clinical entity with only several hundred cases reported in the literature to date [1]. It is defined as a non-traumatic extravasation of urine from the ureter and most commonly results from acute ureteral obstruction, which leads to a sudden increase in intraluminal pressure exceeding the tensile strength of the ureteral wall and causing rupture. Urinary stones represent the most common underlying cause; other less frequent etiologies include ureteral strictures, malignancies, infections, pregnancy, and retroperitoneal fibrosis [2]. In rare instances, no identifiable cause is found despite thorough investigation; these cases are classified as idiopathic or spontaneous ureteral rupture [3]. The symptoms are nonspecific, making diagnosis at the clinical stage unsuspected. Imaging, particularly abdominal CT with contrast injection, is the key examination for diagnosis, allowing precise localization of the rupture and identification of associated complications [4]. Delayed management can lead to severe complications such as urinoma, retroperitoneal abscess, infectious complications with potential septicemia, and even renal failure [5].

We present a case of a 77-year-old patient whose abdominal pain revealed a spontaneous rupture of the left urinary tract in the absence of an apparent etiology.

## Case presentation

A 77-year-old patient, without medical history or surgical interventions, first presented to the emergency department with bloating and intense abdominal pain. The pain appeared spontaneously, without any history of recent or past abdominal trauma, strenuous physical activity, or urinary symptoms such as hematuria or dysuria.

At the first consultation, the patient was clinically stable and was mistakenly treated for presumed gastroenteritis before being discharged home. Due to worsening abdominal pain despite analgesic treatment, the patient returned for a second evaluation.

On physical examination, the patient was conscious, afebrile, and had normal vital signs. The abdomen was tender with guarding in the left iliac fossa; no palpable abdominal mass was noted.

**Figure 1:**
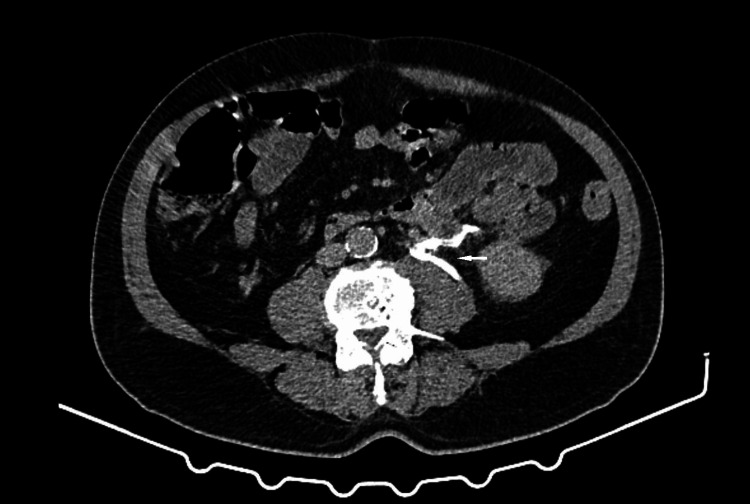
Axial CT image obtained after intravenous contrast administration demonstrating leakage of contrast material from the proximal left ureter, consistent with ureteral rupture.

**Figure 2:**
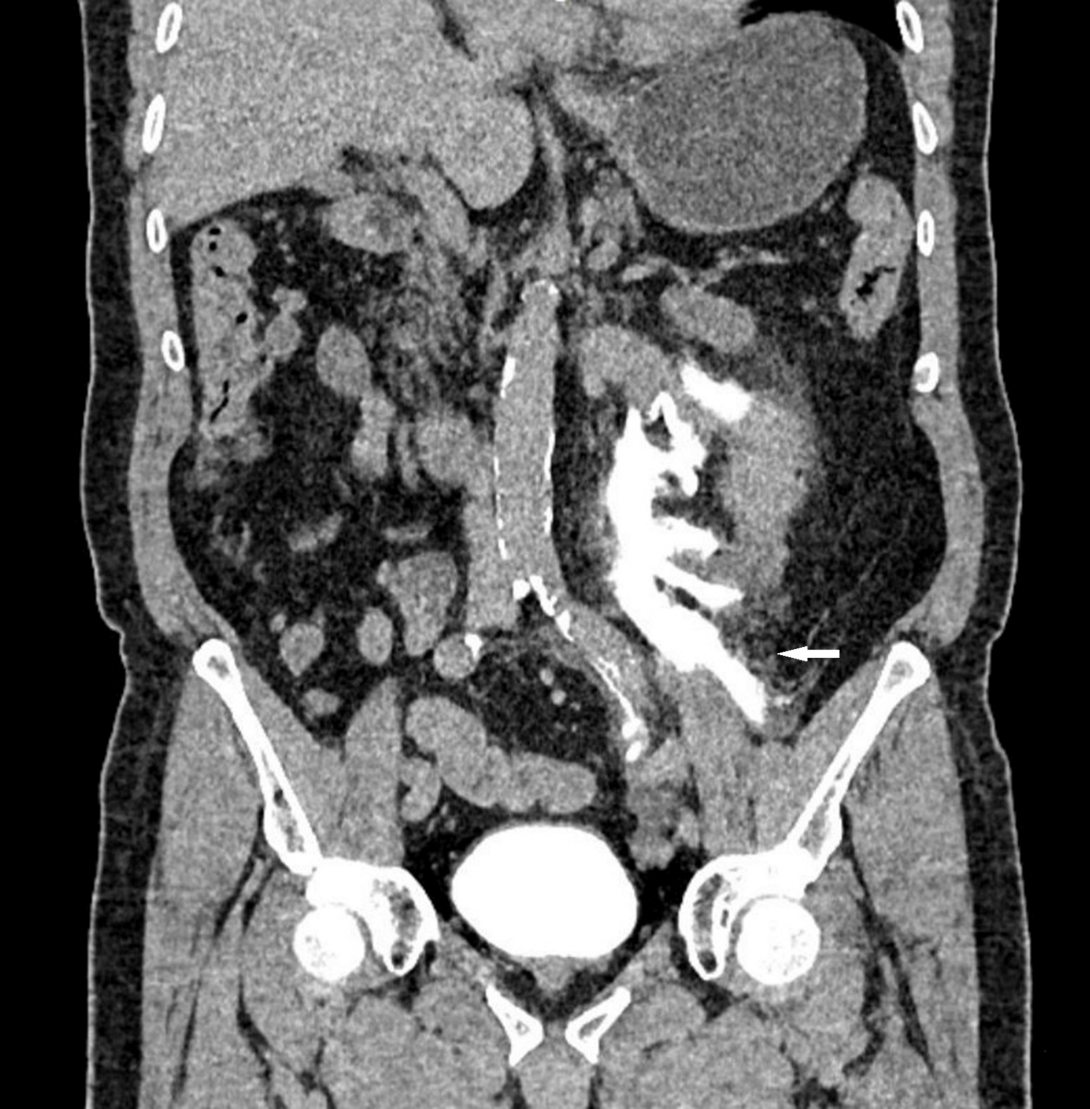
Coronal CT image obtained after intravenous contrast administration demonstrating leakage of contrast material from the proximal left ureter, consistent with ureteral rupture.

Given the persistence of symptoms, the patient’s advanced age, and the unclear clinical presentation, we decided to perform an abdominopelvic CT scan with contrast enhancement, which showed extravasation of contrast in the delayed urinary phase at the level of the superior pole of the left kidney and the ipsilateral ureter, suggesting a rupture of the left urinary tract. There was significant periureteral and left perirenal infiltration. The CT scan did not reveal any factor that could have caused a ureteral rupture, no obstructive urolithiasis, ureteral stricture, tumor, or other identifiable cause of ureteral rupture was detected on imaging (Figures 1, 2).

The diagnosis of spontaneous rupture of the left ureter was explained to the patient, along with the potential complications and the need for surgical or endourological management. Despite detailed information, the patient declined hospitalization and further treatment and chose to leave the hospital against medical advice. Unfortunately, he was subsequently lost to follow-up.

## Discussion

Spontaneous rupture of the ureter is a rare entity; it corresponds to an extravasation of urine from the ureter that occurs in the absence of trauma, prior urological intervention, or known underlying urinary tract pathology [1-3]. In most reported cases in the literature, ureteral rupture is due to an obstructive process, most commonly ureteral calculi, which leads to increased intraluminal pressure [6]. Other reported causes include congenital anomalies, abdominal or pelvic masses, retroperitoneal fibrosis, iatrogenic or post-radiotherapy stenoses, kidney transplantation, and connective tissue diseases [7]. In contrast, our patient presented with a spontaneous ureteral rupture without any identifiable obstructive or predisposing factor on imaging, making this case particularly unusual. Based on our review, to date, fewer than 10 cases of spontaneous ureteral rupture without an identified cause have been reported in the literature over the past two decades.

Similar to previously published cases, our patient presented with acute abdominal pain mimicking an acute abdomen, highlighting the nonspecific clinical presentation of this condition [1-8]. However, unlike most reported cases, which typically involve younger patients or those presenting with flank pain suggestive of renal colic, our patient was elderly and initially misdiagnosed with gastroenteritis, emphasizing the diagnostic challenge in this population.

Rupture may involve any segment of the urinary tract, most commonly affecting the fornix and the proximal ureter [9]. which is consistent with the findings in our case.

Contrast-enhanced computed tomography and urography allow examination of the morphology and function of the urinary tract to detect abnormalities, and are therefore the imaging modality of choice for diagnosis [10]. Our case proves once again the pivotal role of CT imaging in establishing the diagnosis, particularly in atypical clinical presentations.

Given the rarity of cases, there are no formalized recommendations on the optimal treatment; management remains at the discretion of each surgeon and may range from observation and conservative treatment to double-J ureteral stenting, percutaneous nephrostomy, or surgical intervention, including urinary diversion [11]. In most published cases, ureteral double-J stenting was the preferred therapeutic option, with favorable outcomes (Table 1). Unfortunately, in our case, definitive management could not be performed due to the patient’s decision to leave the hospital, which represents a limitation of this report. Nevertheless, the initial diagnostic approach and imaging findings remain valuable for clinical awareness.

**Table 1:** Reports on spontaneous rupture of the ureter without obvious causes over the last 20 years.

Author	Title	Age	Sex	Presentation	Localization	Imaging modality	Main therapy	Outcome
Eken et al. [11]	Spontaneous rupture of the ureter	29	Male	Left flank pain, nausea, and chills	Left upper ureter	Intravenous contrast-enhanced computed tomography	Double-J stenting	Favorable
Aggarwal & Adhikary [3]	Spontaneous ureteric rupture, a reality or a faux pas?	65	Male	Right-sided severe abdominal pain	Right upper ureter	Intravenous contrast-enhanced computed tomography	Double-J stenting	Favorable
Kazaz et al. [8]	Spontaneous rupture of proximal ureter: a case report	65	Female	Left flank pain	Left upper ureter	Intravenous contrast-enhanced computed tomography	Conservative treatment	Favorable
Chiu et al. [12]	A case of spontaneous ureteral rupture mimicking renal colic	67	Female	Left flank pain, nausea, and vomiting	Left upper and mid-ureter	Intravenous contrast-enhanced computed tomography	Double-J stenting	Favorable
Sarbay et al. [13]	Spontaneous ureteral rupture: a case report	24	Male	Left flank pain	Left upper ureter	Intravenous contrast-enhanced computed tomography	Double-J stenting	Favorable

## Conclusions

Spontaneous ureteral rupture is a rare but clinically significant condition that may occur even without identifiable underlying causes. It should be considered in patients presenting with acute flank pain or suspected renal colic or even acute abdominal pain. Contrast-enhanced computed tomography is essential for diagnosis. Early recognition allows appropriate management, prevent potential complications and improve clinical outcome.
